# Antagonistic regulation of the meristemoid-to-guard mother-cell-transition

**DOI:** 10.3389/fpls.2013.00401

**Published:** 2013-10-11

**Authors:** Laura Serna

**Affiliations:** Facultad de Ciencias del Medio Ambiente y Bioquímica, Universidad de Castilla-La ManchaToledo, Spain

**Keywords:** SPCH, MUTE, meristemoid self-renewal, guard mother cell fate, MAPK

The building of a multicellular organism from a single cell is the result of coordinated acquisition of different cell identities in an ordered spatial arrangement. But, how do cells learn about their identity? Stomatal development in Arabidopsis provides perhaps one of the most tractable contexts in which to examine concepts of cell fate determination, as a variety of lineage tracing techniques, including long-term confocal time lapse imaging (Peterson and Torii, 2012), can be deployed in different genetic contexts and environmental conditions to trace the history of only a few cell types. In this essay, I focus on the choice between meristemoid cell self-renewal, in which one of daughters of a dividing meristemoid retains the properties of the parent cell, and its transition through guard mother cell (GMC) fate to produce stomata.

In Arabidopsis, stomatal development, which starts at the tip of the leaf and proceeds basipetally, takes place through a series of stereotyped yet flexible cell division pattern (Lau and Bergmann, 2012; Pillitteri and Torii, 2012). The first sign of stomatal development is an unequal cell division from a protodermal cell called the meristemoid mother cell (MMC). This cell division produces a small triangular meristemoid and a larger neighboring cell. Meristemoids are self-renewing cells that divide (amplifying divisions) in an inward spiral and always yield a larger cell and a smaller meristemoid that maintains its self-renewal activity. The meristemoid eventually loses its self-renewal character and adopts a rounded shape giving rise to the GMC. The GMC undergoes an equal and symmetric cell division that generates the paired guard cells. Some of the larger cells that result from the unequal divisions of MMCs or meristemoids and that come into contact with the stoma (or its precursor) can adopt MMC fate initiating the cell division pattern that culminates with stomatal formation. Others differentiate into pavement cells. This lineage is responsible for generating the majority of the epidermal cells in the leaves (Geisler et al., 2000).

All stomatal lineages originate by an unequal division, but the number of subsequent cell divisions is not fixed, and it varies from zero to three. For example, the study of cell divisions through time using serial imprints revealed that, in the leaves (abaxial side) of C24 ecotype, 44% of complexes develop their stomata prematurely, that is, after the second, or even the first, unequal cell division (Berger and Altmann, 2000). Similar studies in Columbia background (abaxial epidermis of leaves and cotyledons) showed that 66% of their stomata develop also after the first or second unequal cell division (Geisler et al., 2000). Clonal analysis in the leaves (adaxial side) of Landsberg *erecta* ecotype also showed some flexibility with at least 13% of the complexes developing stomata prematurely (Serna et al., 2002). The differences in the degree of flexibility of stomatal development among different studies could be due to the genetic background, the face analyzed, the growth conditions and/or the methodology used to trace stomatal lineages. Anyway, what is certain is that, a greater or lesser extent, the number of amplifying divisions that take place before GMC formation is flexible. More recently, long-term confocal time lapse imaging in Arabidopsis leaves underlined also this variability in the number of amplifying divisions (Robinson et al., 2011). But, what are the molecular bases of this plasticity?

**Figure 1:**
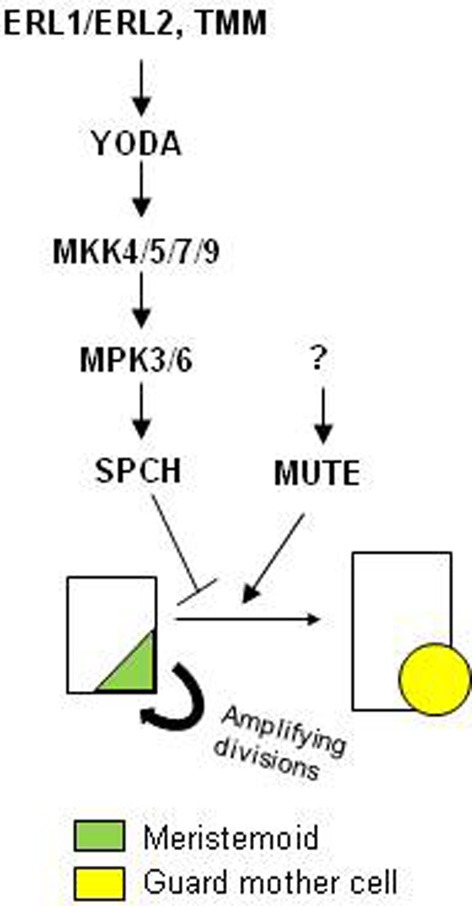
**Model for the regulation of the self-renewal behavior of the meristemoids and their transition to guard mother cell (GMC) fate.** Predominance of SPCH, which is activated by YDA MAPK module, inhibits the meristemoid-to-GMC transition by promoting the self-renewal behavior of the meristemoids. In contrast, predominance of MUTE represses the amplifying divisions of the meristemoids and enhances their transition through the GMC fate. GMC symmetrically divides to produce paired-guard cells (not shown). The components upstream of MUTE remain unknown. SCRM1 and SCRM2 function in this transition through direct interaction with SPCH and MUTE (not shown).

Time-lapse confocal imaging in live leaves showed that the basic helix-loop-helix (bHLH) factor SPEECHLESS (SPCH) locates in MMCs and meristemoids, and disappears in GMCs. This finding strongly suggests a role for SPCH, which drives the cell division that initiates the stomatal-cell lineage (MacAlister et al., 2007; Pillitteri et al., 2007), in the maintenance of meristemoid self-renewal activity in the leaves (Robinson et al., 2011). Agree with this interpretation, and suggesting that the proposed role of *SPCH* in leaves extends to other plant organs, meristemoids of the pedicel epidermis of the weak *spch-2* mutant undergo significantly fewer amplifying divisions compared with wild type plants (MacAlister et al., 2007). In addition to SPCH, MAPKs also provide flexibility to stomatal development through repression of the meristemoid-guard mother cell switch (Lampard et al., 2009): constitutive activation, beginning in meristemoids, of either the mitogen-activated protein kinases (MAPK) kinase kinase YODA (YDA) or the MAPK kinases MKK4, MKK5, MKK7 or MKK9 prevents stomatal formation giving rise to an epidermis consisting of pavement cells and arrested meristemoids. Because both MKK4 and MKK5 function upstream of the MAP kinases MPK3 and MPK6 during stomatal development (Wang et al., 2007), it is expected that constitutive activation, beginning in meristemoids, of MPK3 or MPK6 also blocks GMC formation. If this is true, and given that MPK3 and MPK6 phosphorylate SPCH protein *in vitro* (Lampard et al., 2008), these phosphorylation events may activate SPCH function in meristemoids, promoting their self-renewal behavior and so repressing their transition to guard-mother cell (Figure 1). This scenario contrasts with that in which SPCH hyper-phosphorylation by both MPK3 and MPK5 suppresses its activity preventing the first unequal cell division of stomatal development (Lampard et al., 2008).

The *ERECTA*-family leucine-rich repeat receptor-like kinases members *ERECTA-LIKE1* (*ERL1*) and *ERECTA-LIKE2* (*ERL2*) maintain the division activity of the meristemoids and prevent them from differentiating into GMCs (Shpak et al., 2005): *erl1*, *erl2* and *erl1 erl2* mutants show a reduction in the number of larger cells that surround the stoma (or its precursor), which arise from amplifying divisions. Like *ERL1* and *ERL2*, both the subtilisin-related extracellular protease *STOMATAL DENSITY AND DISTRIBUTION1* (*SDD1*) and the plasma membrane-anchored leucine-rich repeat receptor-like protein *TOO MANY MOUTHS* (*TMM*) have been also involved in the maintaining of the self-renewal activity of the meristemoids, with *sdd1-1* and *tmm* mutants undergoing also less amplifying divisions than wild-type plants (Berger and Altmann, 2000; Geisler et al., 2000; Bhave et al., 2009). Because TMM associates with ERL1 *in vivo* (Lee et al., 2012), the formation of heterodimeric complexes between TMM and ER1, and most probably between TMM and ERL2, might be required for the initiation of this signaling cascade. Moreover, the fact that constitutive activation of YDA suppresses the defects of *tmm* places this extracellular signaling upstream of YDA (Bergmann et al., 2004). It is known that these receptors interact and are activated by peptide ligands of the EPIDERMAL PATTERNING FACTOR (EPF)/EPIDERMAL PATTERNING FACTOR-LIKE (EPFL)-family (Torii, 2012). However, at the moment, no peptide ligand has been directly involved in the self-renewal activity of the meristemoids.

*MUTE* also encodes a bHLH protein, which, in addition, exhibits high homology with SPCH (Pillitteri et al., 2007). The loss-of-function *mute* mutant lacks stomata but develops meristemoids that arrest after excessive amplifying cell divisions that take place in an inward-spiral pattern (MacAlister et al., 2007; Pillitteri et al., 2007). This suggests that *MUTE* represses self-renewal activity of the meristemoids and induces GMC formation. Both *MUTE* promoter activity and the MUTE protein localization are restricted to a subset of meristemoids (MacAlister et al., 2007; Pillitteri et al., 2007), which presumably will undergo GMC transition. Interestingly, MUTE does not seem to be a substrate of either MPK3 or MPK6 (Lampard et al., 2008). Even more, MUTE is phosphorylated *in vitro* by the MAP kinase MPK4. However, both loss-of-function and gain-of-function of the MAPK kinase kinase MEKK1, which acts upstream of MPK4 (Asai et al., 2002), do not induce any effect on stomatal development (Wang et al., 2007). In addition, the expression, beginning in meristemoids (or MMCs), of constitutively activate MAPK kinase kinase MKK1 or MKK2, which phosphorylate MPK4 (Popescu et al., 2009), also confers no apparent effect on the control of stomatal development, including meristemoid self-renewal activity (Lampard et al., 2009). Then, if MPK4 phosphorylates MUTE in meristemoids, this phosphorylation event should not be correlated with the MUTE function in the control of the meristemoid-to-guard mother-cell-transition. If this is true, both loss- and gain-of-function of MPK4 plants should exhibit no effect on stomatal development.

Together, these finding suggest that at least two parallel pathways control the switch from meristemoid to GMC (Figure 1):(1) a pathway that controls SPCH function, and (2) an unknown pathway that regulates the activity of MUTE. Predominance of MUTE pathway may repress meristemoid self-renewal behavior, accelerating stomata formation. In contrast, predominance of SPCH pathway may induce the opposite effect, promoting self-renewal behavior of the meristemoids, and delaying GMC formation. In addition, RT-PCR analysis showed that SPCH promotes its own transcription and the transcription of *MUTE* (MacAlister et al., 2007; Pillitteri et al., 2007). Genetic and biochemical data also suggest that both SCRM1 and SCRM2 function in this transition through direct interaction with SPCH and MUTE (Kanaoka et al., 2008).

Stomatal number, and so gas exchange with the atmosphere, depends on the number of MMCs formed and on how long it takes for GMCs to divide. Since stomata premature formation reduces the distance among neighboring stomata, and vice versa, a coordinated control of self-renewal of meristemoids and their transition to GMC fate is fundamental to avoid maladaptive responses. Meristemoids may perceive signals, adjusting the levels of SPCH vs. MUTE activity, in order to mount physiologically appropriate developmental responses. But, how could these signals trigger the regulation of SPCH and/or MUTE activity? Agree with the general view that MAPK modules are integrating point of multiple signals, several studies have highlighted that YDA-MAPK module is a point of integration for communicating brassinosteroids and light signaling to stomatal development (Kang et al., 2009; Kim et al., 2012; Khan et al., 2013). Brassinosteroids, through the glycogen synthase kinase 3-like kinase BRASSINOSTEROID INSENSITIVE 2, can also regulate SPCH activity directly (Gudesblat et al., 2012), which, most probably, takes place in situations that trigger a reduction of YDA-MAPK levels (Serna, 2013). ABA also controls stomatal development (Tanaka et al., 2013), and YDA-MAPK module may also serve as a transmitter of the ABA-dependent cascade. Certainly, MPK6 is a target of ABSCISIC ACID-INSENSITIVE 1 (Leung et al., 2006), which represses stomatal formation (Tanaka et al., 2013). Considering that YDA-MAPK module controls the meristemoid to GMC transition, it is likely that these factors also regulate this switch. MUTE may also perceive, directly or indirectly through their yet unknown upstream regulators, systemic signals to optimize the ratio of stomata vs. pavement cells to the state of the plant and the environment.

Interestingly, analysis of static pictures showed that the number of meristemoids and GMCs is not constant throughout development (Geisler and Sack, 2002). Instead, there are periods when, for example, both meristemoids and GMCs increase and decrease, respectively (Geisler and Sack, 2002). This suggests that, in this particular period, meristemoids receive signals that accentuate the predominance of SPCH pathway, thus prolonging their self-renewal capacity. There are also periods when meristemoids drop, while GMCs increase (Geisler and Sack, 2002), suggesting, in this period, a predominance of MUTE pathway in these meristemoids, even though they had divided a variable number of times. Agree with this interpretation, data from serial imprints showed that in some temporal windows the majority of meristemoids become mature stomata, even though they do it prematurely (Geisler and Sack, 2002). Meristemoids also pause divisions when subject to mild osmotic stress, and quickly recover them once this stress is alleviated (Skirycz et al., 2011). Mild osmotic stress may block MUTE activity, which in absence, or under low levels, of SPCH activity, would pause meristemoid progression. Selective activation of either SPCH or MUTE may resume meristemoid self-renewal capacity or its transition to GMC fate, respectively. Supporting this interpretation, arrested meristemoids of 13 days-old *iMUTEmute* plants transit to GMC fate upon late (13 days post-germination) induction of β-estradiol-dependent *MUTE* expression (Triviño et al., 2013).

The story gets more complicated when we consider stomatal development in the context of the entire developing leaf, instead of considering individual lineages. For example, if two meristemoids from two separate lineages arise adjacent to each other, they do not progress to become stomata, but instead one of them undergoes an additional cell division to prevent the physical contact with its neighbor, or even undergoes a transdifferentiation process (Geisler et al., 2000). This example highlights that cell-to-cell signaling guides meristemoid fate, most probably, by imposing to that dictated by environmental factors or phytohormones.

Detailed knowledge about the process that regulates self-renewal of meristemoids and their transit to GMC fate is crucial to enable better adapted and more productive plants to local site conditions. To deep into the understanding of the control of this step is necessary to identify the components acting upstream of MUTE. Challenges for the future also include verifying that MPK3 and MPK6 are downstream components of the four MAPK kinases, MKK4, MKK5, MKK7, and MKK9, in the meristemoid to GMC transition, and that MPK3 and MPK6 trigger SPCH regulation in this switch. Identifying the components upstream of TMM-ERL1/2 is also needed.
